# GMEnzy: A Genetically Modified Enzybiotic Database

**DOI:** 10.1371/journal.pone.0103687

**Published:** 2014-08-01

**Authors:** Hongyu Wu, Jinjiang Huang, Hairong Lu, Guodong Li, Qingshan Huang

**Affiliations:** 1 State Key Laboratory of Genetic Engineering, Institute of Genetics, School of Life Sciences, Fudan University, Shanghai, China; 2 Shanghai Engineering Research Center Of Industrial Microorganisms, Shanghai, China; 3 Shanghai High-Tech United Bio-Technological R&D Co., Ltd., Shanghai, China; University of Lausanne, Switzerland

## Abstract

GMEs are genetically modified enzybiotics created through molecular engineering approaches to deal with the increasing problem of antibiotic resistance prevalence. We present a fully manually curated database, GMEnzy, which focuses on GMEs and their design strategies, production and purification methods, and biological activity data. GMEnzy collects and integrates all available GMEs and their related information into one web based database. Currently GMEnzy holds 186 GMEs from published literature. The GMEnzy interface is easy to use, and allows users to rapidly retrieve data according to desired search criteria. GMEnzy’s construction will increase the efficiency and convenience of improving these bioactive proteins for specific requirements, and will expand the arsenal available for researches to control drug-resistant pathogens. This database will prove valuable for researchers interested in genetically modified enzybiotics studies. GMEnzy is freely available on the Web at http://biotechlab.fudan.edu.cn/database/gmenzy/.

## Introduction

The need to develop new classes of antibiotics with novel mechanisms of action against drug-resistant pathogens is urgent, given the increasing prevalence of antibiotic resistance [1]. Thus, the Infectious Diseases Society of America (IDSA) has launched a new collaboration titled the “10 X ‘20” initiative to develop ten new antibacterial drugs by 2020 [2]. Enzybiotics, in light of their capability to kill susceptible organisms when applied exogenously as recombinant proteins, and because of their low probability for inducing bacterial resistance, have attracted much attention as potential substitutes for conventional antibiotics [3]–[6].

Enzybiotics primarily consist of endolysins and virion-associated peptidoglycan hydrolases from bacteriophages, bacteriocins and autolysins from bacteria, and lysozymes [7]–[9]. A number of recent studies have demonstrated the strong potential enzybiotics have for controlling and treating infection caused by varied pathogens, especially gram-positive bacteria [7], [10]–[19]. However, enzybiotics when applied externally, often require modification to improve their properties in specific applications, particularly in complex environments such as matrices, blood, or on mucous membranes [13]. Several molecular engineering techniques, including domain shuffling, sequence truncation, and random or rational mutagenesis, have been used to optimize enzybiotics by altering their binding or lytic capabilities [13], [20]. Among these methods, domain exchange is unique to enzybiotics because of their modular architecture [10], [21]. In the early 1990s, Diaz et al. were the earliest to do this, and demonstrated that shuffling functional domains between the pneumococcal autolysin LytA and the phage lysine Cpl-1, switches the catalytic activity and binding properties of the resulting chimeric enzymes [22]. We refer to these types of enzybiotics as genetically modified enzybiotics (GMEs).

In general, enzybiotics are considered antimicrobial peptides on the basis of their protein nature and antibacterial activities. In fact, enzybiotics are different from antimicrobial peptides in both molecular size and antimicrobial mode of action. Although many databases [23]–[25] have been developed devoted to antimicrobial peptides, there are presently only two existing databases that focus on enzybiotics: EnzyBase [26] and phiBIOTICS [27]. The former contains 1144 enzybiotics from 216 natural sources, whereas the latter represents the knowledge encompassing 21 enzybiotics and 69 corresponding research studies. Moreover, no existing database provides information on how GMEs can be designed, produced, or obtained. With this observation, we developed the manually curated database, GMEnzy.

GMEnzy focuses on GMEs and stores not only basic GME information but also design strategy, production and purification method, and activity data. GMEnzy will serve as a unique tool for GME studies. The construction of GMEnzy will enhance understanding the mechanism of GME action, and increase the efficiency and convenience of developing these bioactive proteins on demand. Eventually, GMEnzy could help reduce and/or delay increases in antibiotic resistance worldwide.

## Results and Discussion

### Database Description

GMEnzy was created as a useful resource for genetically modified enzybiotics studies. As a web based database, GMEnzy provides a user-friendly web interface for users to easily query and retrieve information on GMEs. All the data in GMEnzy can be accessed and retrieved directly from a web browser. All data were manually collected from scientific literature and public databases, or were calculated by computer programs. All GMEnzy data are categorized and stored in relational tables using MySQL.

**Figure 1:**
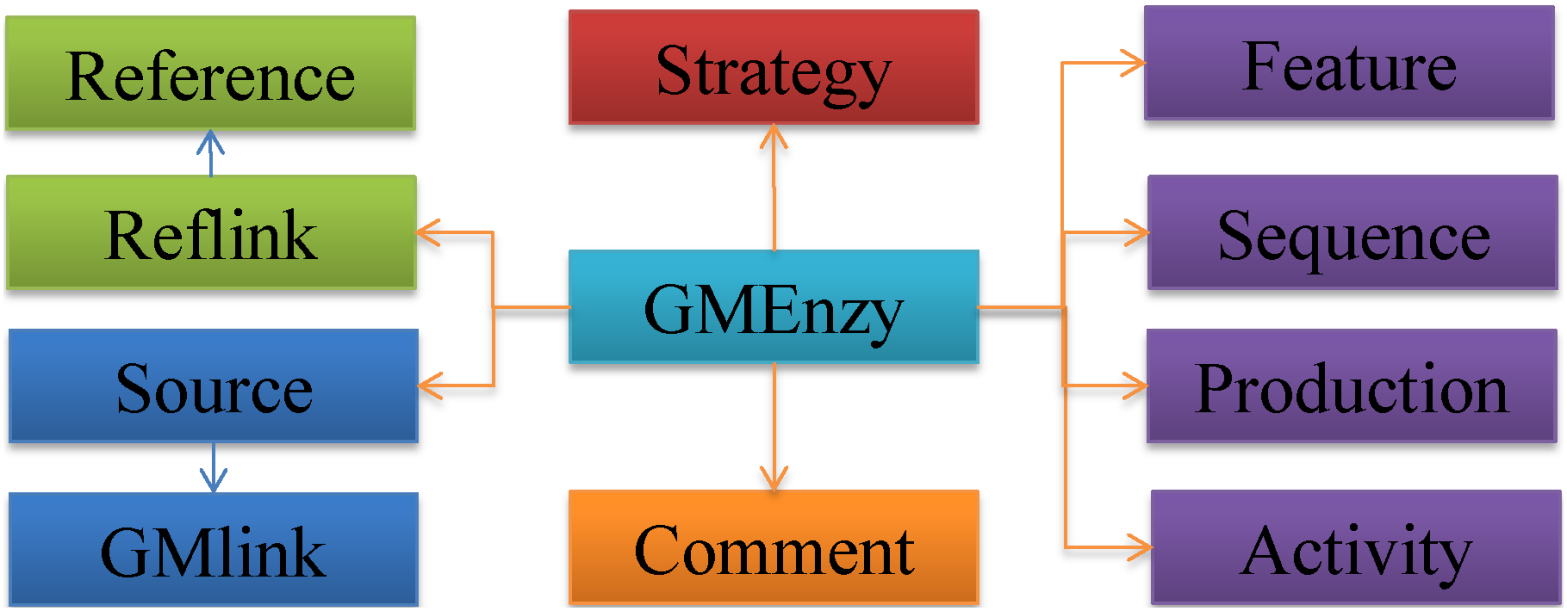
Schema of the GMEnzy database.

GMEnzy comprises eleven MySQL relational tables. Figure 1 shows the schema of the database. The table GMEnzy contains the protein definition, function, and physiochemical properties information fields. Reference information related to each GME is stored in Reference and Reflink. Information related to creating and producing GMEs is contained in Strategy and Production respectively. The features of each GME and its annotation are included in Feature. The parent enzybiotics which GMEs derived from and their annotations are involved in Source and GMlink respectively. Available protein sequences are contained in the table Sequence. Antimicrobial activity data *in vitro* or *in vivo* for each GME are stored in Activity. Users can add comments to any of the GMEs, and these comments are added to the Comment table.

### Database Interfaces

**Figure 2:**
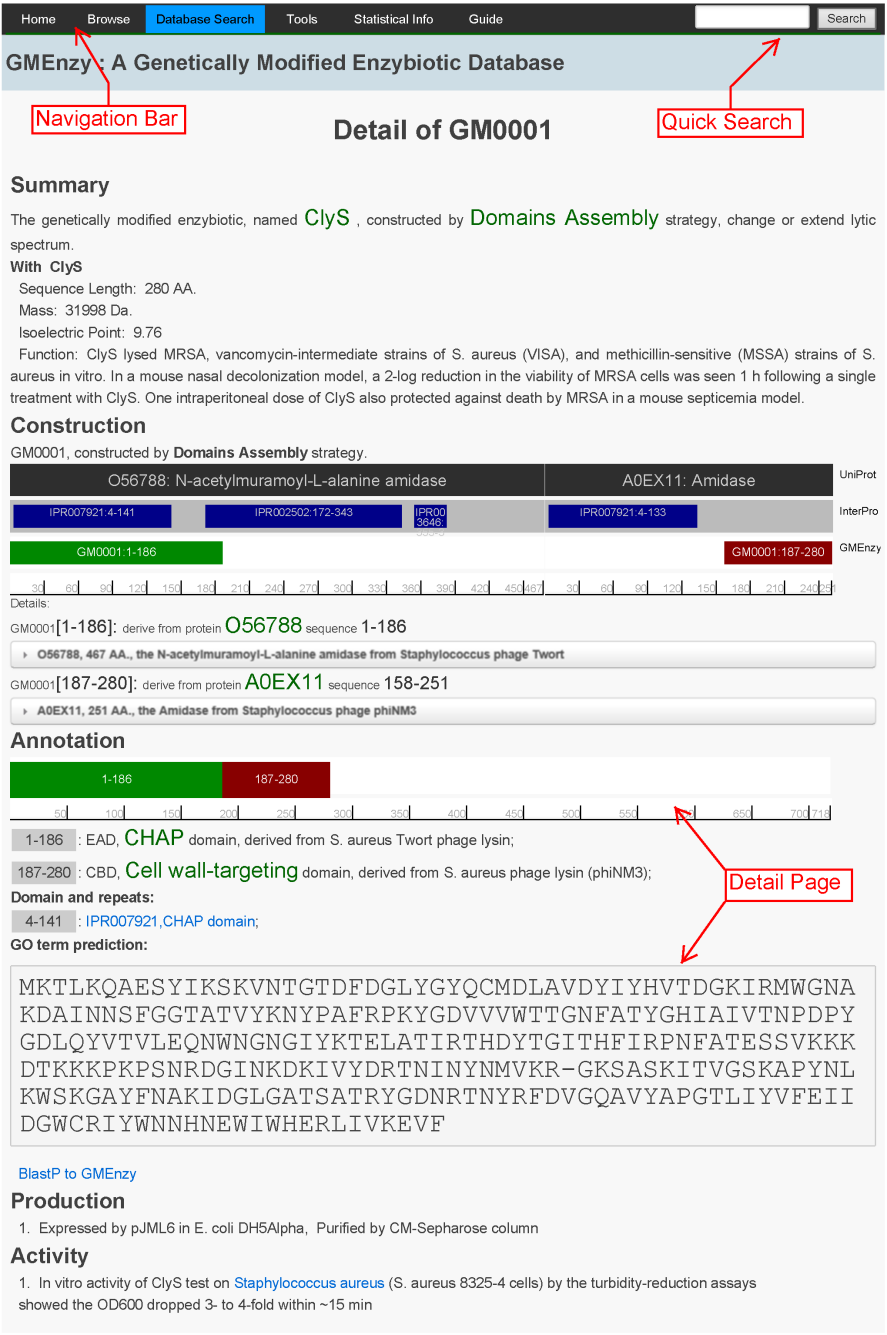
Screen shots of a GME result page. The screen shots show the advanced search and result views. Please note that not all fields are shown.

A concise navigational interface that contains the database Browse, Search, Tools, Statistical Info, and Guide options was designed to generate a clearly structured database layout that enables fast and easy navigation (Figure 2).

The Browse interface allows users to navigate not only the entire database, but also grouped GMEs classified by specified creation strategies. Moreover, the Browse interface contains an additional link that allows the download of all GMEs in FASTA format.

The Search interface can be used to retrieve specific information using either a Quick or Advanced option. A Quick search only allows keywords, while the Advanced search accepts the specification of up to six separate fields: GM Id, protein name, parent enzybiotic name, modified strategy, domains, and target organism. The user can query the database by either one particular condition or a combination of various conditions. In addition, the search result page provides the function for quick alignment of the results by the ClustalW [28] software when the results more than two. Each GME Search produces a results page with seven sections: summary, construction, annotation, production, activity, references, and comments (Figure 2). Summary information consists of enzybiotic name, construction strategy, and resulting effect. In addition, summary information also provides basic physiochemical properties such as sequence length, calculated protein mass and isoelectric point (pI), and simple functional annotation. Construction information demonstrates how the GME derived from parent proteins and its correlation with functional alterations. The details information describes the parent protein which the GME derived from and the links to databases describing them. The annotation section demonstrates all components within each GME along with brief descriptions and lists function annotations and the deduced sequence. Moreover, a link to BlastP againt GMEnzy was added below the sequence. Production, activity, and reference information related to each GME is also listed below. Furthermore, a comment form at the bottom of the page provides users the option to submit comments for the displayed GME to facilitate the exchange of information among researchers.

The Tools interface implements the ClustalW and BlastP functions. The ClustalW function provides a simple ClustalW service for users to alignment the GME by ClustalW software and a link to EBI ClustalW which supplied more options for multiple sequence alignment. The BlastP function provides only the BLASTP against GMEnzy service and a link to NCBI BLASTP for users who want to blast full datasets in NCBI.

The Statistical info interface supplies statistical data on the physiochemical properties, production, and effect information of the GMEs. (see ‘Statistical description and findings’ section below for further details).

The Guide interface provides simple instructions to potential users on how to use GMEnzy functions.

### Statistical Description and Findings

**Figure 3:**
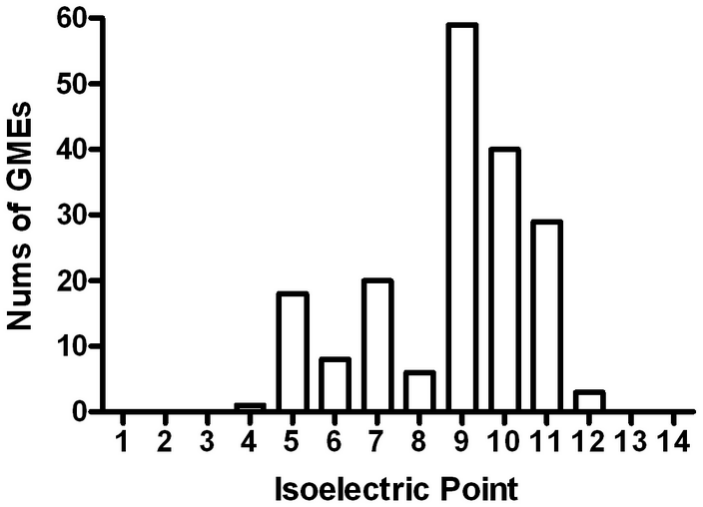
Distribution of calculated isoelectric points for the GMEs in GMEnzy. Every bar indicates the number of GMEs calculated to have their isoelectric point range from pI-1 to pI.

The current version of GMEnzy contains 186 GMEs. Most of GMEs in GMEnzy are novel sequences. We used BLASTP [29], [30] to align full length GME against UniProt [31], and only 17 sequences showed identical hits from UniProt. The lengths of the GME sequences range from 35 to 879 amino acids. The distribution of pIs for all the GMEs is shown in Figure 3. The majority (98.4%) of the GMEs have calculated pIs that range from 5 to 11, with most of these being basic, rather than acidic.

**Figure 4:**
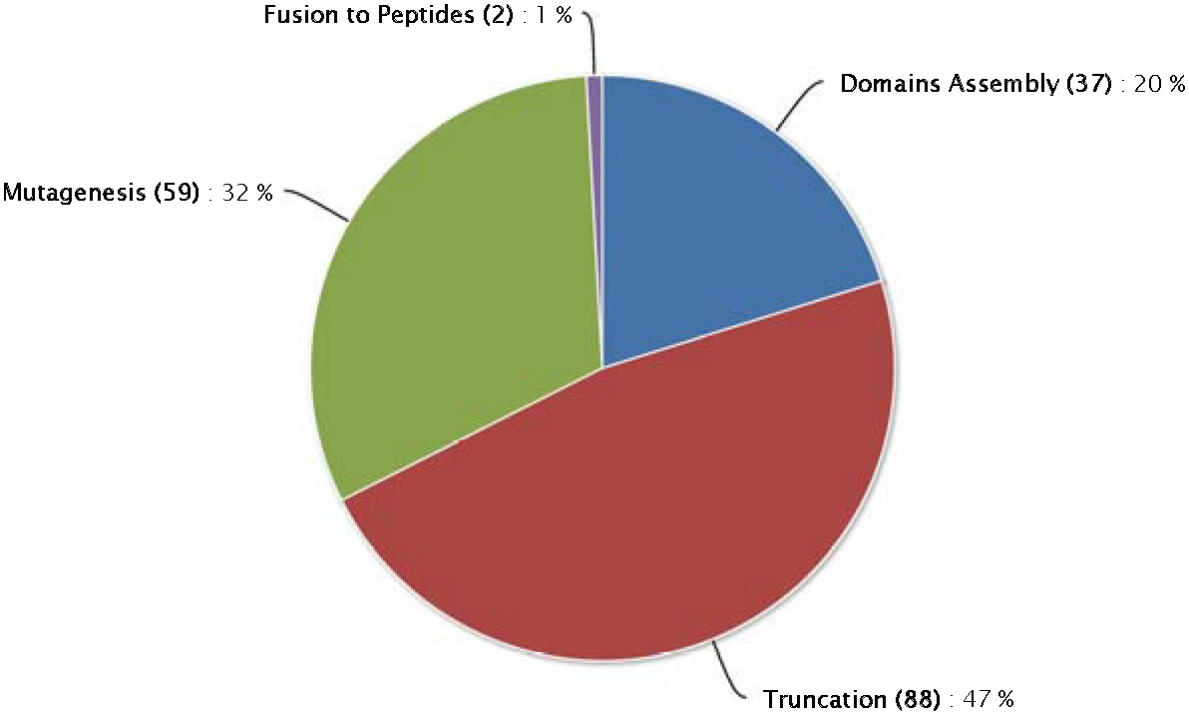
Methods for GME construction.

Presently molecular engineering approaches for GME construction mainly include domain exchange, truncation, random and/or rational mutagenesis, and fusion to peptides, although specific modification methods are varied [13]. Thus, we categorized all GMEs into four groups by construction strategy. Figure 4 demonstrates these categories and the proportion of GMEs in each. In GMEnzy the truncation and mutagenesis method accounts for most (79%, 147/186) of the cases. The domain assembly method includes 20% (37/186) of the cases, while the fusion to peptides modification only contains 1% (2/186) of the cases (Figure 4). This distribution is in accord with the current state of GME research. In the past GMEs were mainly created for the control of gram-positive bacterial infections. More recently the fusion to peptide approach was designed as a new orientation for killing gram-negative pathogens. Furthermore, Díez-Martínez et al. have introduced an efficient method for improving the lethal effect of Cpl-7 by inverting the net charge of its cell wall-binding module [20]. This technique could be adopted to help enzybiotics fight gram-negative pathogens. Finally, about 27% (50/186) of the entries in the database are GMEs that exhibit ideal or improved properties, such as increased lytic activity or an extended lytic spectrum, after modification (Table 1). Our data suggest that among these approaches, domain assembly modification exhibits 91% (34/38) positive results, and is the best method for genetic modification (other than the fusion to peptides technique, which has only two cases).

**Table 1:** Strategies for GME construction and their resulting effect.

Strategy	Effect	Nums of GMEs	Positive rate (%)*
Domains assembly	**Change or extend lytic spectrum**	**18**	91
	**Improve the lytic activity**	**10**	
	**Enhance the solubility, change or** **extend lytic spectrum**	**2**	
	Lose the activities of cell targeting	1	
	**Enhance the solubility**	**1**	
	Decrease the lytic activity	1	
	Keep the lytic activity	1	
Trunction	Decrease the lytic activity	38	16
	**Improve the lytic activity**	**14**	
	Lose the activities of cell targeting	14	
	Lose the lytic activity	8	
	Keep the lytic activity	7	
	Keep the activities of cell targeting	6	
	Undisclosed	2	
Mutagensis	Decrease the lytic activity	26	5
	Keep the lytic activity	25	
	Decrease the binding activity	4	
	Change or extend lytic spectrum	2	
	Lose the activities of cell targeting	2	
	Keep the activities of cell targeting	1	
	**Improve the lytic activity**	**1**	
Fusion to peptides	**Change or extend lytic spectrum**	**2**	100

*, positive rate indicates the proportion of GMEs with improving lytic activity or extending lytic spectrum (with bold font) to all calculated GMEs.

**Figure 5:**
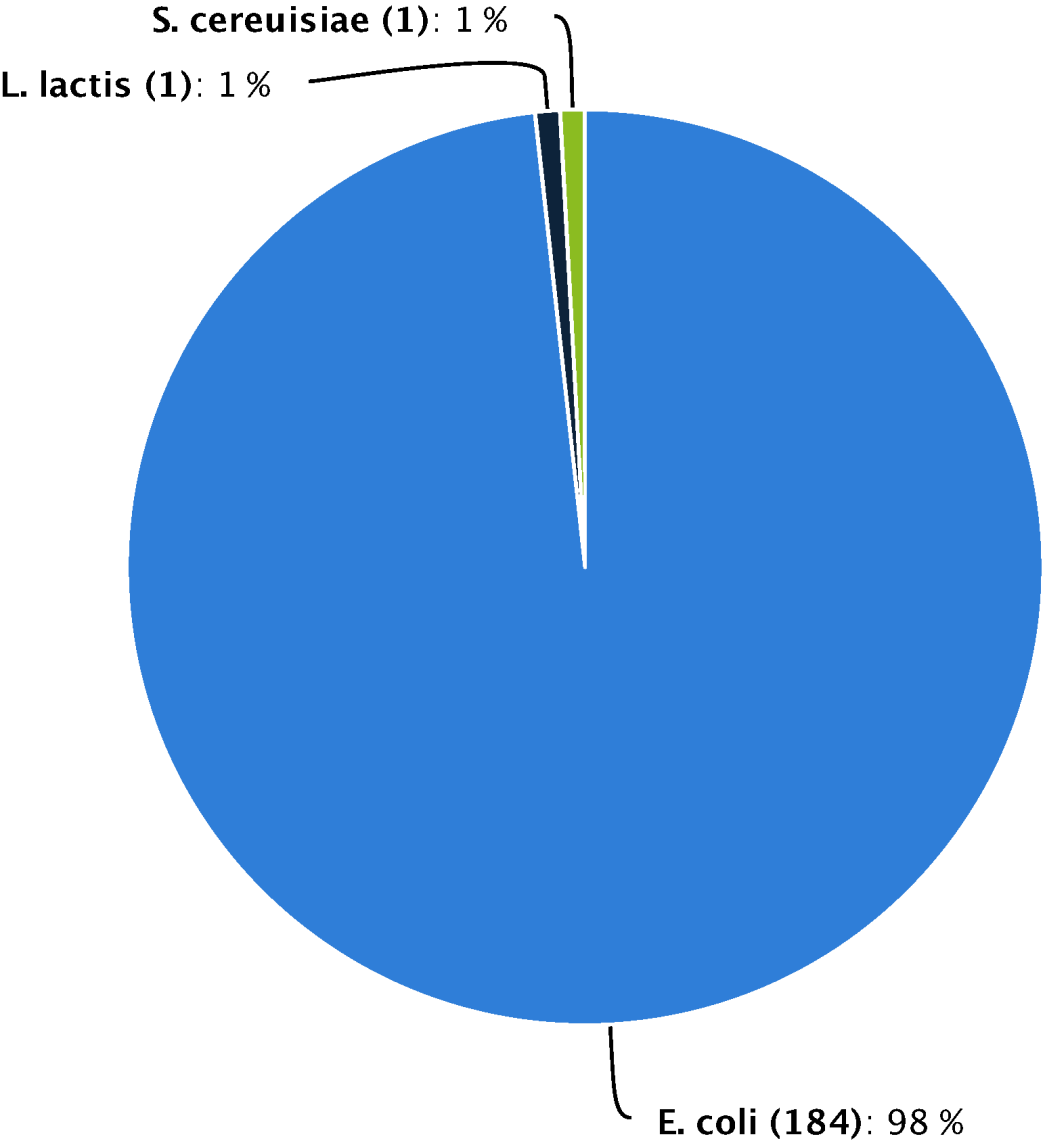
Hosts for producing GMEs.

**Figure 6:**
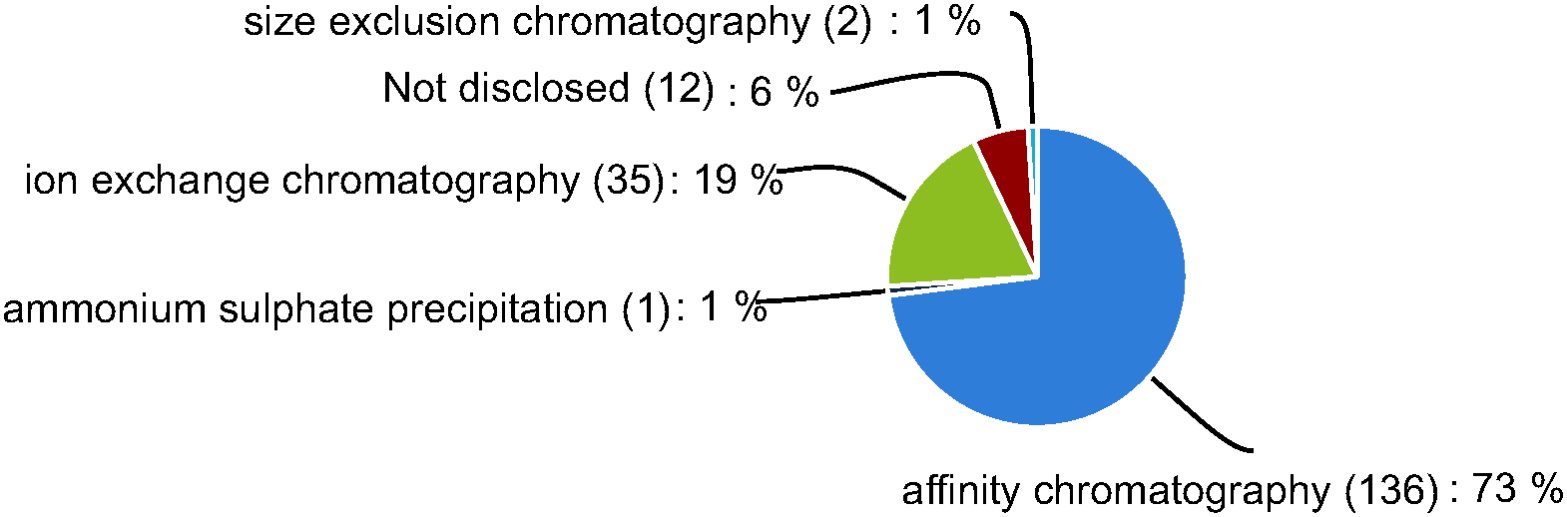
Purification methods for GMEs product.

Bacteria are the most commonly used expression host, according the data in our database. Bacteria are hosts in more than 99% of all cases, while only one case used *S. cerevisiae* (Figure 5). The bacterium *E. coli* is the most popular expression host because of attractive features of the organism itself (e.g. fast growth, low cost, and convenient operation). Additionally, to facilitate rapid purification, most (73%, 136/184) of the GMEs are normally expressed as fusion proteins with either histidine (His) or glutathione-S-transferase (GST) purification tags (Figure 6), as well as data not disclosed. Ion-exchange chromatography and size exclusion chromatography purification techniques represent 19% (35/184) and 1% (2/184) of all GMEs respectively. The traditional ammonium sulfate precipitation method has almost been abandoned, as only one case in our database uses it to purify a GME.

### Limitations and Future Prospects

Currently GMEnzy only holds 186 GMEs. Moreover, because of rapidly emerging drug-resistance in bacteria, GMEs have attracted increasing interest as potential antimicrobial agents. Therefore, available data will rapidly increase. We will collect each new GME’s activity information and related data and incorporate them into GMEnzy by updating the database twice a year in the future.

## Conclusions

GMEnzy is a web accessible database that focuses on the genetically modified enzybiotics. The current version of GMEnzy has 186 entries (as of January, 2014). The database can be queried either by simply using keywords or by combinatorial conditions searches. GMEnzy will not only aid in expanding our current understanding of GMEs and their mechanisms of action, but may have implications in the development of new GMEs to fight against increasingly drug-resistant pathogens. GMEnzy is now available at http://biotechlab.fudan.edu.cn/database/gmenzy/.

## Materials and Methods

### Data Collection and Organization

We collected all available GME information from scientific literature by searching the Web of Science and Google scholar with the following query: (keyword: “hydrolyse” OR “endolysin” OR “lysine” OR “lysozyme” OR “enzybiotics” AND “fusion” OR “chimeric”), and also by exploring the references listed in the literature discovered with this first pass. Each citation returned from the search was further checked manually to assure it contained the desired information. All GMEs abstracted from the literature were classified into four groups based on the protein engineering strategies: domain assembly, truncation, mutagenesis, and fusion to peptides. All GME sequences were constructed from the UniProt database according to descriptions from the literature. Additional physicochemical data for each GME was either calculated via Bioperl programs or obtained from the literature. All of the collected information was classified and filled into eleven relational tables in MySQL. GMEnzy currently contains 186 GMEs acquired from more than 100 published papers.

### Web Interface and Application

GMEnzy was built on a 64 bit Windows (2008 R2) server running WAMPSERVER (V2.2d), which integrates the Apache HTTP Server (V2.2.21) with PHP (V5.3.10) and the MySQL Server (V5.5.20). All entries are stored in a MySQL database. The application was coded in PHP, using the jQuery JavaScript Library (V1.6.2), the Highchart jQuery Plugin, and Cascading Style Sheets (CSS) for the web design. Apache, MySQL, PHP, and jQuery were preferred as they are open-source software and platform independent, making them suitable for academic use. The web server and all parts of the database are hosted at the Information Office of Fudan University, Shanghai, China.
